# Implant-prosthetic rehabilitation of patients with severe horizontal bone deficit on mini-implants with two-piece design—retrospective analysis after a mean follow-up of 5 years

**DOI:** 10.1186/s40729-021-00353-8

**Published:** 2021-07-28

**Authors:** Lukas Wimmer, Pantelis Petrakakis, Karim El-Mahdy, Surian Herrmann, Dirk Nolte

**Affiliations:** 1 Clinic for Oral & Maxillofacial Surgery mkg-muc®, Munich, Germany; 2 Private Dental Practice, St. Johann, Salzburg, Austria; 3 Private Practice, Düsseldorf, Germany; 4 Department of Restorative Dentistry & Periodontology, Dental School, Ludwig Maximilian University, Munich, Germany; 5 Ruhr University Bochum, Bochum, Germany

**Keywords:** Dental implant, Mini-implants, Two-piece implants, Diameter-reduced implants, Severe horizontal bone atrophy, Patient-related outcome measures, Patient satisfaction

## Abstract

**Background:**

As a consequence of tooth loss due to trauma or extraction, a reduced alveolar crest volume limits the deployment of standard implants in certain patient cases. For this reason, minimal-invasive treatment with mini-dental implants (MDI) might be an option to allow implant treatment even in cases with severe horizontal bone loss without augmentation measures. The aim of this retrospective cohort study was to investigate clinical and radiological implant, as well as patient-related parameters after treatment with MDI.

**Results:**

Clinical and radiological records of 19 female (82.6%) and 4 male patients (17.4%) (*N* = 23), who received 52 mini-dental implants with a two-piece design in a single surgical center between November 2011 and October 2018, were retrospectively analyzed. Implants were submitted to conventional loading on different types of screwed superstructures. Crestal bone loss was measured on standardized periapical radiographs. Patient-related outcome parameters (PROMs) were recorded during follow-up period. Mean clinical and radiological follow-up was 69.6 months (5.8 years) and 51.6 months (4.3 years), respectively. Three implants were lost in two patients, leading to an implant survival rate of 94.2%. Mean radiological crestal bone loss was 1.6 mm. Both amount of peri-implant recession and crestal bone loss were significantly correlated (*r* = 0.65; *p* < 0.001). Likewise, a significant correlation was observed between deeper probing depths and increased peri-implant bone loss (*r* = 0.41; *p* = 0.012). Alveolar ridges with a reduced alveolar crest width were significantly correlated with higher peri-implant bone loss as well (*r* = − 0.33; *p* = 0.011). No prosthetic complications were reported during follow-up. Extent of midfacial recession and papilla height loss had a significant negative impact on most of the PROMs.

**Conclusions:**

Treatment with MDI seems to be a successful alternative treatment option, especially for elderly patients with reduced crest width at implant sites. Due to the good clinical results and high survival and success rates, this treatment option was associated with high patient satisfaction. Despite the promising results, particular consideration should be given to appropriate treatment planning in these patients due to the strong correlation between peri-implant soft-tissue parameters, crestal bone loss, and reduced alveolar crest width.

## Background

Implant treatment is increasingly gaining interest for many patients, who are looking for alternative options to conventional prosthetic treatment in cases of dental aplasia or after tooth loss in terms of better aesthetical and functional results. Due to the observation that bone remodelling does not result in a uniform resorption pattern of the alveolar ridge after tooth extraction, standard implant dimensions were one of the most common constraints for implant treatment in cases of severe bone loss for many years. A pronounced horizontal bone loss up to 50.0% of the original width of the alveolar ridge was observed within 12 months after tooth extraction [1].

As successful treatment with standard-diameter implants requires a sufficient alveolar crest width of at least 6.0 mm and an adequate space between natural teeth in case of single space implant placement, many attempts were made in order to create dental implants with a reduced diameter, thus allowing a wider range of indications for implant treatment [2–5].

Based on first observations, displaying excellent osseointegration results after placement of orthodontic or transitional small diameter implants for stabilization of temporary prosthetic superstructures, treatment with diameter reduced mini-implants came into focus of scientific investigation, and debate as a viable substitute to treatment with standard implants [4]. Long-term use of implants with a reduced diameter < 3.0 mm were licensed by the Federal Drug Administration (FDA) first-time in 1997.

Further beneficial effects of this new treatment option were lower expenses, a reduced treatment time by avoiding extensive bone augmentation procedures, and a minimal-invasive surgical approach without the need of full thickness flap elevation, thus minimizing or even avoiding potential post-operative complications [4].

A considerable number of systematic reviews display no significant differences in mean crestal bone loss [6–9] and survival rates [6, 8–11] between standard and mini-implants.

Most clinical studies on reduced diameter implants < 3.0 mm were performed with one-piece, screw-shaped titanium implants, which were usually applied in dental implantology for many years [5, 12]. Only a small number of clinical studies reported on reduced diameter implants with a two-piece design [5, 13–15].

As shown by the growing number of qualitative studies over the last 20 years, patient reported outcome parameters (PROMs) are increasingly moving into the focus of dental implantology. Insights on patient expectations and motivation for implant treatment are relieved by the use of questionnaires on patient satisfaction, oral health-related quality of life aspects, respectively. These insights facilitate patient counseling, as well as patient-centered decision-making on an individual level in dental practice [16].

For this reason, the present study was performed to investigate clinical performance of two-piece implants with reduced diameter after a mean clinical follow-up of 5.8 years and a mean radiological observation period of 4.3 years under the clinical condition of severe horizontal alveolar bone loss. In order to measure PROMs, a self-created questionnaire was applied at the end of the radiological follow-up.

## Methods

In this retrospective clinical study, clinical and radiological records of consecutive outpatients were analyzed. All patients were referred by their dentists between November 2011 and October 2018 to our surgical center for implant-treatment.

Patient’s age and a reduced horizontal ridge dimension were defined as relevant criteria for the decision for treatment with MDI. Due to the retrospective design of the study, no pre-selection of patients according to distinct inclusion or exclusion criteria was performed.

Patients were provided with two-piece, diameter-reduced implants (mini-dental implants, MDI) due to marked localized or generalized atrophy of the alveolar ridge. After cessation of the surgical part of implant treatment, prosthetic treatment was performed by the referring dentists.

The main hypothesis of the present retrospective study was that treatment with two-piece MDI without application of extensive augmentation procedures comprises comparable results with respect to implant survival/success rates, crestal bone loss, and patient satisfaction, as reported with standard implants in literature.

**Fig. 1:**
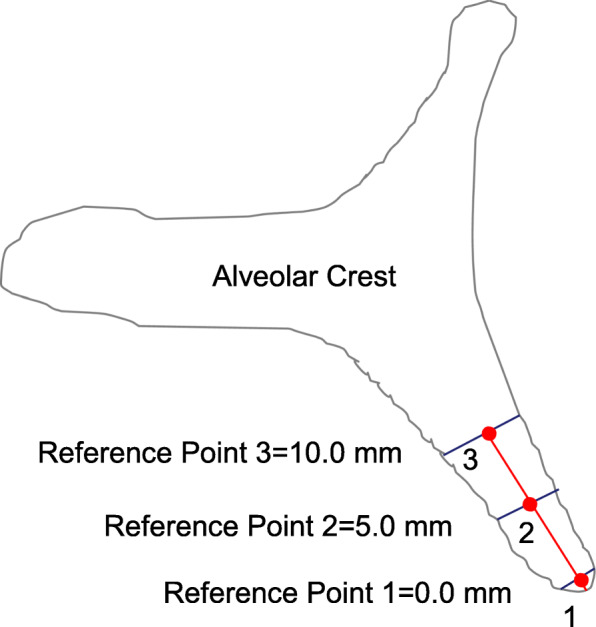
CBCT measurements of the alveolar crest at implant site

Three-dimensional (3D) cone beam computer tomography (CBCT) measurements were performed pre-operatively at three levels of implant sites (equicrestally and 5.0 mm, 10.0 mm subcrestally, respectively), in order to obtain sufficient information on alveolar crest volume, as well as a basis for a 3D determination of the implant position (Fig. 1).

**Fig. 2:**
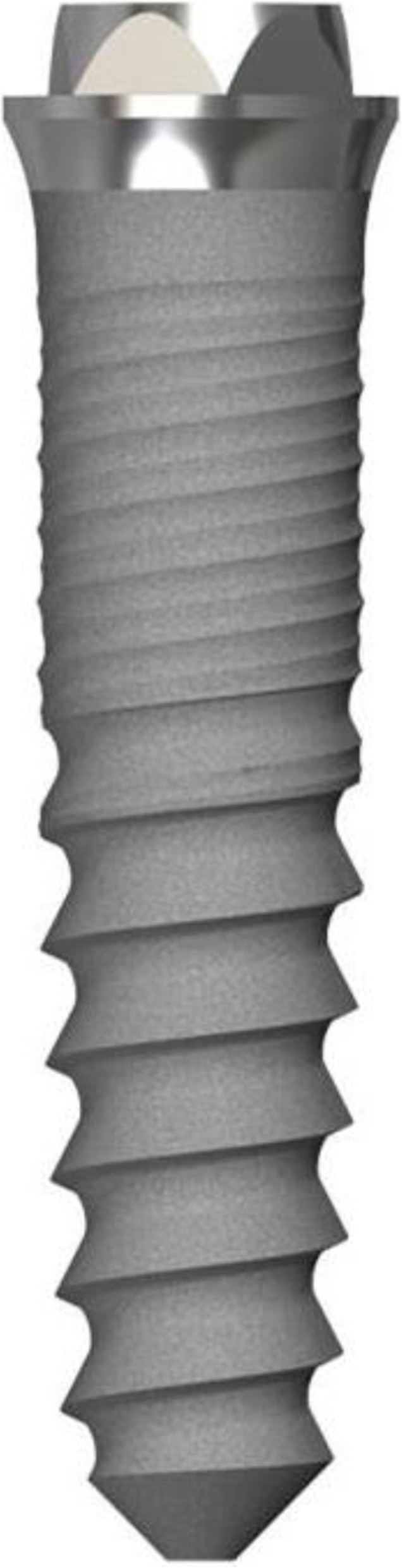
Design of the utilized implant system

The root-shaped MDI used in our investigation are made of grade 4 titanium and comprise a homogenous, highly purified surface. Implants have a bone condensing design, in order to receive a sufficient primary stability in early healing phases by compression of the peri-implant bone. Implants comprise a micro-grooved collar design and an external hexagonal connection, which shall facilitate placement of tilted abutments for adjustment of potential implant angulations (Fig. 2). MDI are currently available in three different diameters (2.7 mm, 2.9 mm, and 3.1 mm), and three lengths (11.5 mm, 13.0 mm, 15.0 mm; BEGO Implant Systems®, Bremen, Germany). As intended by the manufacturer, only MDI with a diameter of 3.1 mm are provided with Locator®-like prosthetic abutments (Easy-Con Mini).

Mini-implants were inserted equicrestally in healed ridges of the maxilla and mandible according to the manufacturer’s surgical protocol, using drills and instruments of the BEGO Mini-/OsseoPlus tray. All implants were placed freehand with a slight deflection of a mucoperiosteal flap in the upper and lower jaw, and without elevation of the sinus membrane, when placed in the posterior parts of the maxilla. Contour augmentation procedures were performed in case of reduced width of the alveolar crest at implant sites either with autologous bone, which was harvested during implant-preparation, or with a xenogenous bone mineral (Bio-Oss® Collagen, Geistlich Biomaterials GmbH, Baden-Baden, Germany).

All subjects were asked to comply with a pharmacological regimen of amoxicillin (3 × 500 mg TID for 7 days) or, if allergic to penicillin, clindamycin tablets (3 × 300 mg TID for 7 days), and analgetic medication (Ibuprofen 600 mg, every 6–8 h as needed to a maximum of 1800 mg/day).

Implants were either submitted to a submerged healing protocol or they were provided with the native cover screws of the implant system. Prosthetic treatment was performed for all implants with a conventional loading protocol after a healing period of 3 months. Panoramic radiography was taken immediately after surgical treatment for assessment of the implant´s location. Assessment of peri-implant bone level was performed at the end of follow-up with apical radiography in a parallel technique (Orthophos, Sirona, Bensheim, Germany), to facilitate a faithful implant-visualization, thus avoiding overlay or distortion effects, as usually observed in panoramic radiographies.

Written informed consent was obtained from all study participants according to the ethical guidelines of the Declaration of Helsinki (Version 2013). Ethical approval was obtained by the ethical committee of the Ludwig-Maximilian University of Munich, Germany (19-255).

All clinical and radiological measurements were performed by a calibrated dentist (LW). Initial clinical soft tissue parameters were recorded by using the periodontal probe as recommended by the community periodontal index of treatment needs (CPITN) [17]. Changes in peri-implant soft tissue height and probing depths, as well as any signs of bleeding/suppuration or implant mobility were recorded at the end of clinical follow-up.

**Fig. 3:**
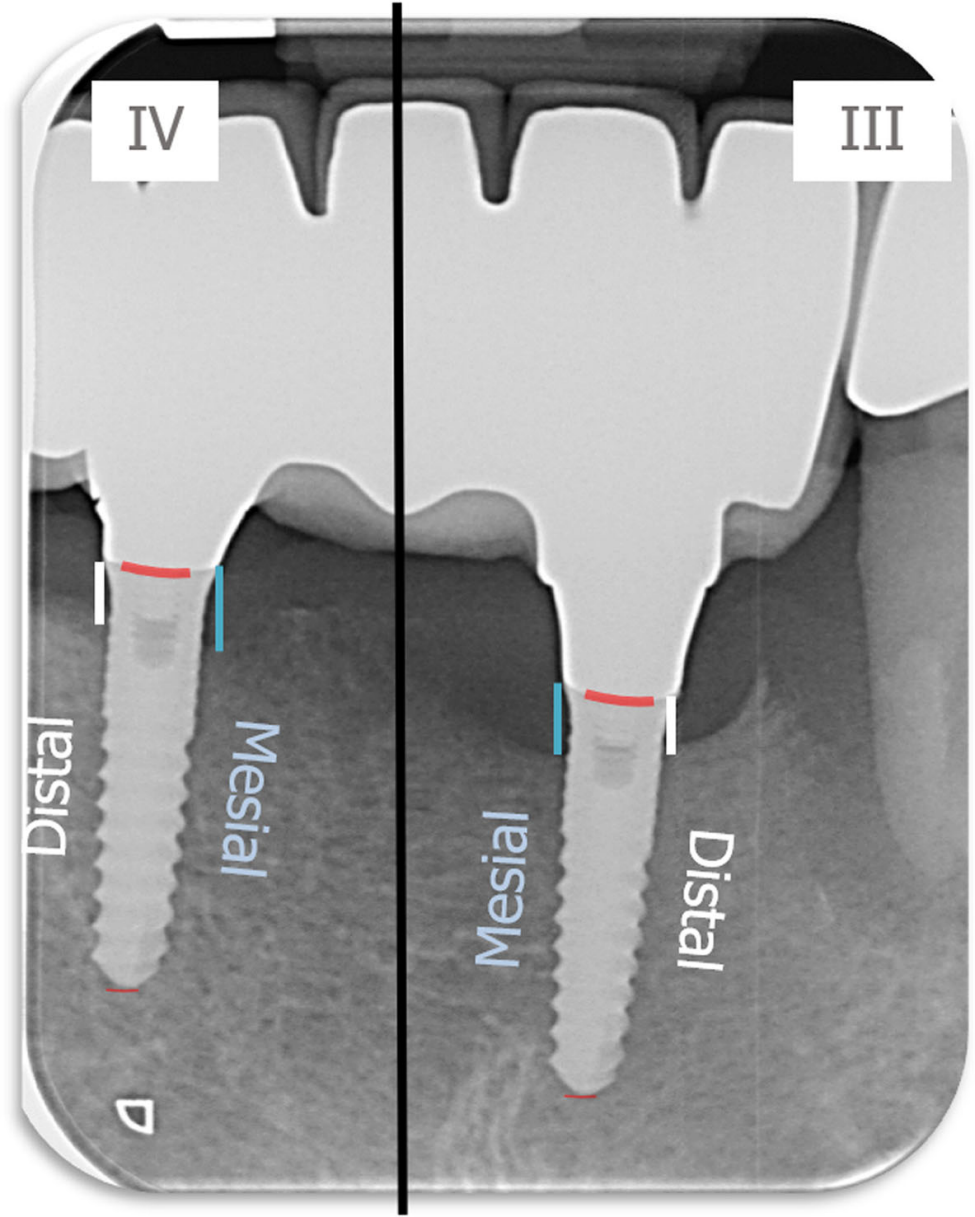
Radiological measurement of crestal bone level

Measurement of crestal bone loss was performed digitally with the Sidexis XG-software (Dentsply Sirona, Bensheim, Germany). Implant shoulder served as reference for the linear measurements mesially and distally of the implant. Bone loss was measured from the most mesial and distal point of the implant shoulder to the deepest crestal point of the peri-implant bone (Fig. 3).

All patients were provided with fixed, screw-retained single crowns, bridges, or full-arch restorations by their referring dentists.

The following criteria of Buser et al. [18] were used to evaluate implant success:

1. Absence of persistent subjective complaints, such as pain, foreign body sensation and/or dysesthesia.
2. Absence of a recurrent peri-implant infection with suppuration.
3. Absence of mobility.
4. Absence of a continuous radiolucency around the implant.
5. Possibility for restoration.

Prosthetic maintenance was performed at every follow-up appointment by assessment of prosthetic fit and functionality.

Absence of persistent subjective complaints, such as pain, foreign body sensation, and/or dysesthesia was retrieved in combination with different PROMs with a self-developed 10-item questionnaire at the end of the follow-up period. The items 2 and 3 (absence of a recurrent peri-implant infection with suppuration, absence of mobility) were retrieved during clinical inspection, while item number 4 was recorded by radiological analysis. Item number 5 was not recorded, due to the two-piece design of the implant, providing the possibility of compensation of improper implant angulation. Patient satisfaction was retrieved by the following parameters:

1. Surgical result.
2. Surgical success.
3. Implant aesthetics.
4. Aesthetics of prosthesis.
5. De novo decision for implant treatment.
6. Recommendation of implant treatment.

Scoring was following the German school grades scale, ranging from grade 1 for the best outcome, full consent, respectively. A scoring with the grade 6 represented the worst result, no consent, respectively.

At the last clinical follow-up examination, patients answered the questionnaire after being carefully instructed by the dental surgeon (LW), who applied a standardized instruction protocol. The surgeon additionally attended the patient throughout the answering process, in order to clarify potential ambiguities during completion of the questionnaire.

Sample calculation was performed with G*Power 3.1.9.2. Based on a one-sided dependent *t* test, with a given effect size of 0.8, a level of significance of 0.05, and a statistical power of 0.95, a sample size of at least 19 patients was calculated. With 23 participants, the requirements of sample size were fulfilled in the present investigation.

Statistical analysis was performed with the MS Excel AddIn Winstat, version 2012.1.0.96 (Robert K. Fitch) and BiAS for Windows (epsilon-Verlag), version 11.10. Test for normal distribution was performed with the Kolmogorov-Smirnov test statistics. In case of parametric/non-parametric distribution of values, either parametric testing (paired and unpaired *t* test) or non-parametric testing was performed (Mann-Whitney *U* test). Spearman rank correlation tests and Pearson correlation tests were applied for analysis of correlations between two variables. Kruskal-Wallis *H* tests were used for multiple group comparison. Chi^2^ test and Fisher’s exact test were utilized for analysis of bivariate data. Level of significance was set at α = 0.05.

## Results

### Patients

Clinical and radiological records of 23 patients were available for data analysis after a mean clinical follow-up of 69.6 months (5.8 years), and a mean radiological follow-up period of 51.6 months (4.3 years). Nineteen patients were female (82.6%) while only four patients were male (17.4%). Mean age was 60.2 years, and there was no gender-specific significant difference in mean age between both groups (men 56.3 ± 27.9 years; women 61.1 ± 16.9 years; *p* = 0.324). With one patient being 18 years old, and another patient being 85 years old at time of implant placement, patient group revealed a high range in age. The majority of patients (*n* = 17; 73.9 %) was ≥ 60.0 years old, with a main distribution in the age class ranging between 60.0 and 69.0 years (*n* = 11; 47.8%). Six patients (26.1 %) were reporting regularly intake of medication like Simvastatin, Ramipril, L-Thyroxin, or ASS. Only two patients (8.7%) reported regularly smoking habits. None of the patients revealed signs of ongoing periodontitis, reported history of former periodontitis respectively. Due to the small number of male patients and patients with smoking habits or regularly intake of medication, further statistical analysis of group-specific differences concerning these independent variables was not performed.

### Mean horizontal alveolar crest width

**Fig. 4:**
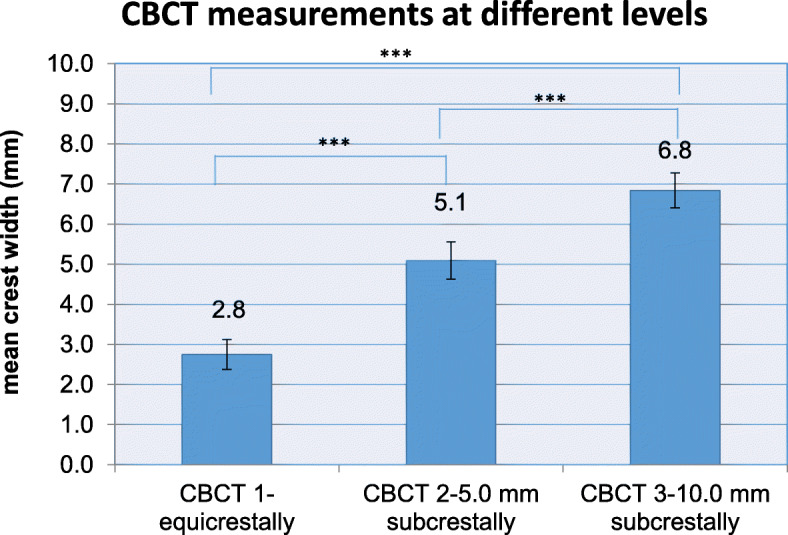
Pre-operative CBCT measurements at three different levels of the alveolar crest displaying significant differences in ridge width

Pre-operative CBCT enabled measurements of the alveolar ridge width at implant sites at three different levels. Mean crestal width revealed 2.8 mm at level 1 (equicrestally), 5.1 mm at level 2 (5.0 mm subcrestally), and 6.8 mm at level 3 (10.0 mm subcrestally), leading to an overall mean crest width of 4.9 mm. Mean alveolar crest width differed significantly between all measurement levels (*p* < 0.001) (Fig. 4).

### Implants and prosthetic reconstructions

Fifty-two MDI with two different diameters and three different lengths were placed in the maxilla and/or in the mandible of the 23 patients with a conventional insertion protocol.

Nineteen of the 52 implants (36.5%) were placed in the maxilla. The majority of implants were placed in the anterior region (12 implants, 63.2%), while the remaining seven implants were inserted in molar areas. Eighteen of the 33 implants, which were inserted in the mandible (54.5%) were placed in the posterior region (12 in premolar and 6 in molar area), whereas the other 15 implants were placed in anterior area.

Additional contour augmentation was performed in 39 implants (17 patients) with autogenous bone (15 implants; 38.5 %) and xenogenous bone material (24 implants; 61.5%) to obtain a sufficient width of peri-implant hard-tissue coverage. The majority of implants (*n* = 47; 90.4%) was submitted to a submerged healing, whereas five implants (9.6%) were left to heal in an open mode. Implant distribution ranged from one single implant to a maximum of six implants per patient. Male patients received in total five implants (9.6%), while female patients were provided with 47 implants (90.4%).

During clinical and radiological follow-up, three implants (5.8%), placed in two patients, were lost due to an increased clinically detectable mobility and signs of peri-implant inflammation, like bleeding and suppuration on probing, thus revealing an implant survival rate of 94.2%. One of the lost implants was placed in one patient in order to close the single gap in region 46. Early implant loss occurred 2 months after implant placement during non-submerged healing. The other two implants were placed in the anterior part of the edentulous maxilla of the second patient for stabilization of an overdenture. Both implants were lost 4 years after insertion.

**Fig. 5:**
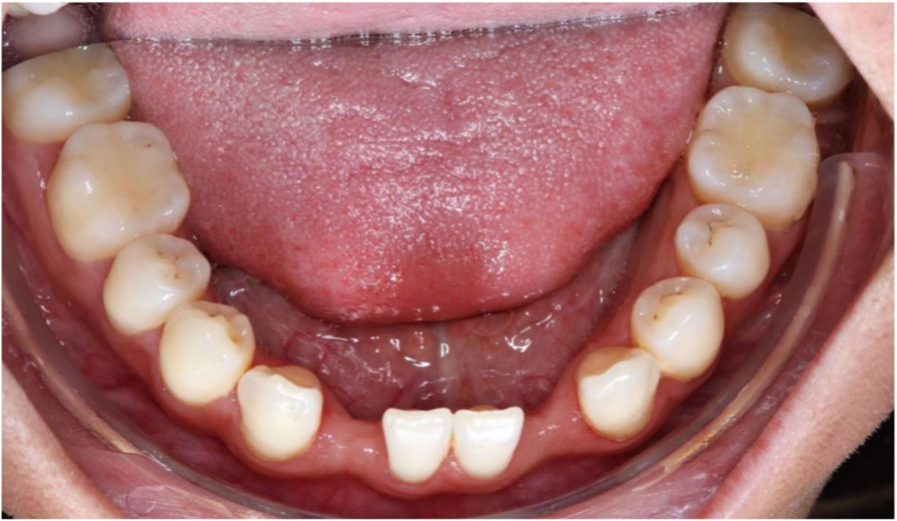
Young patient with two congenitally missing lateral incisors in the mandible

**Fig. 6:**
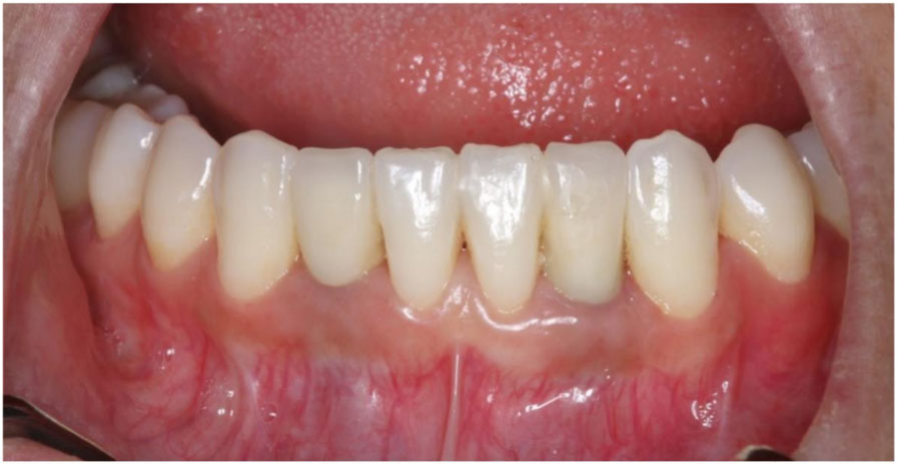
Same patient after implant-prosthetic treatment with single crowns at clinical follow-up 1 year post-OP

**Fig. 7:**
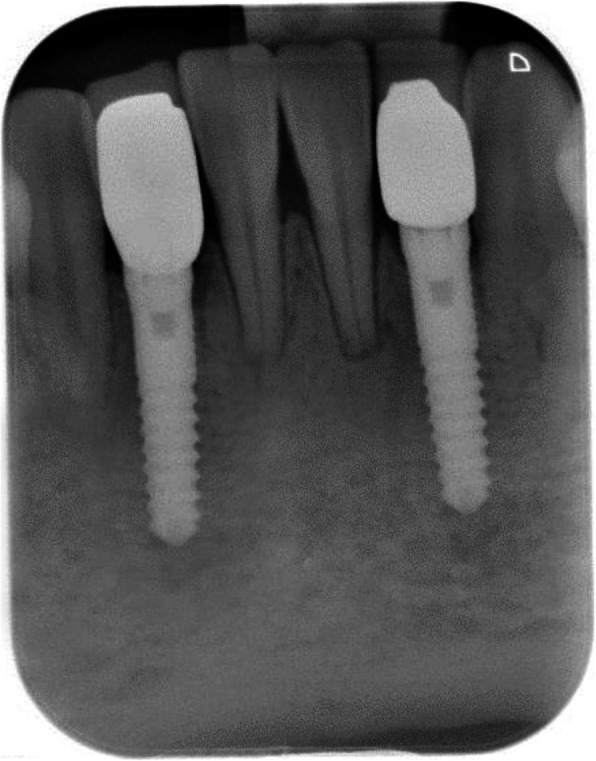
Radiological follow-up of the same patient 1 year post-OP

**Fig. 8:**
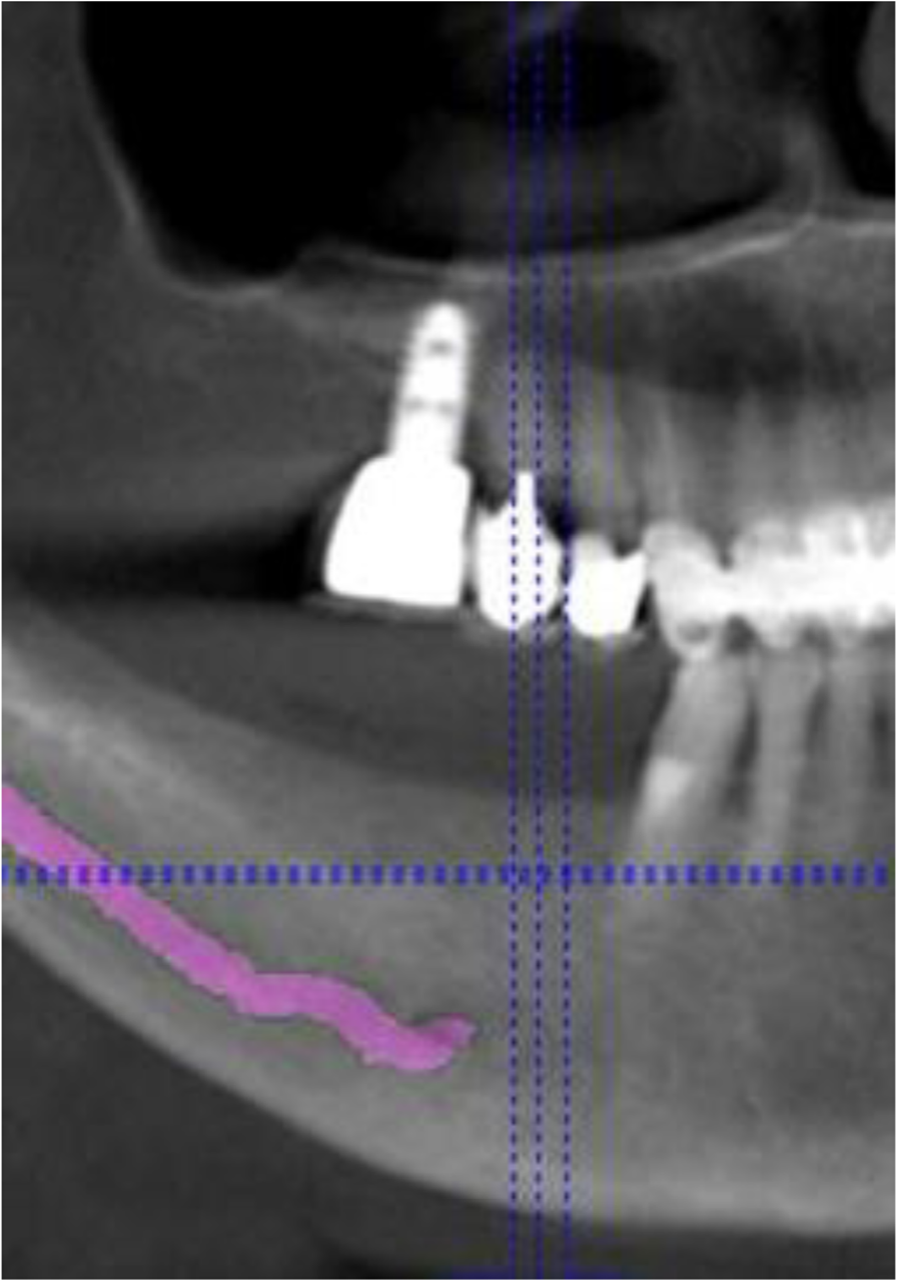
CBCT for pre-OP surgical planning of an elderly female patient (76 years) with severe horizontal bone deficit in the right mandible with missing teeth 44, 45, 46, and 47. The inferior alveolar nerve is visualized in pink color

**Fig. 9:**
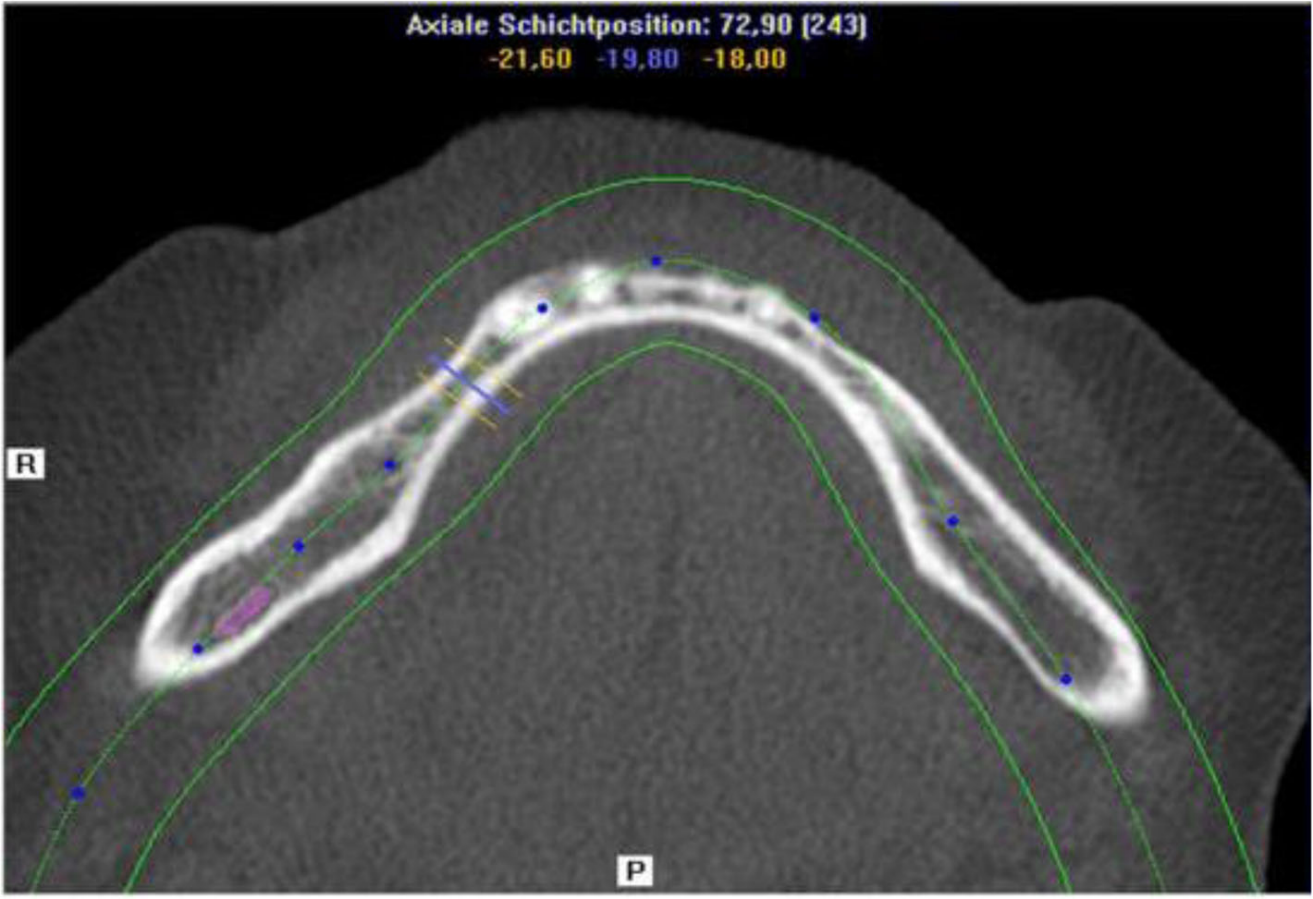
Horizontal section showing bony deficit at implant sites

**Fig. 10:**
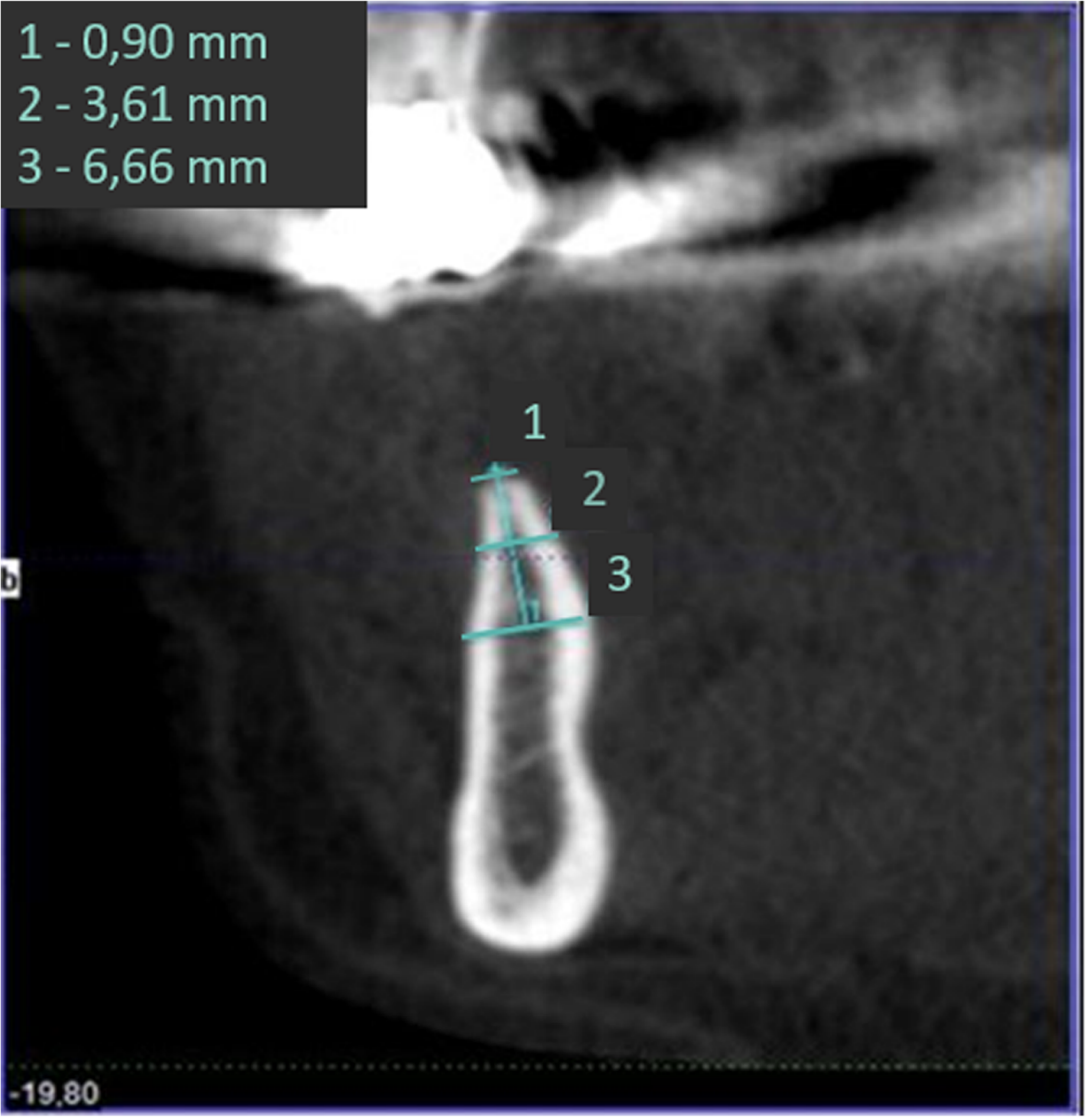
Sagittal section at implant site with markings at reference points (see Fig. 1)

**Fig. 11:**
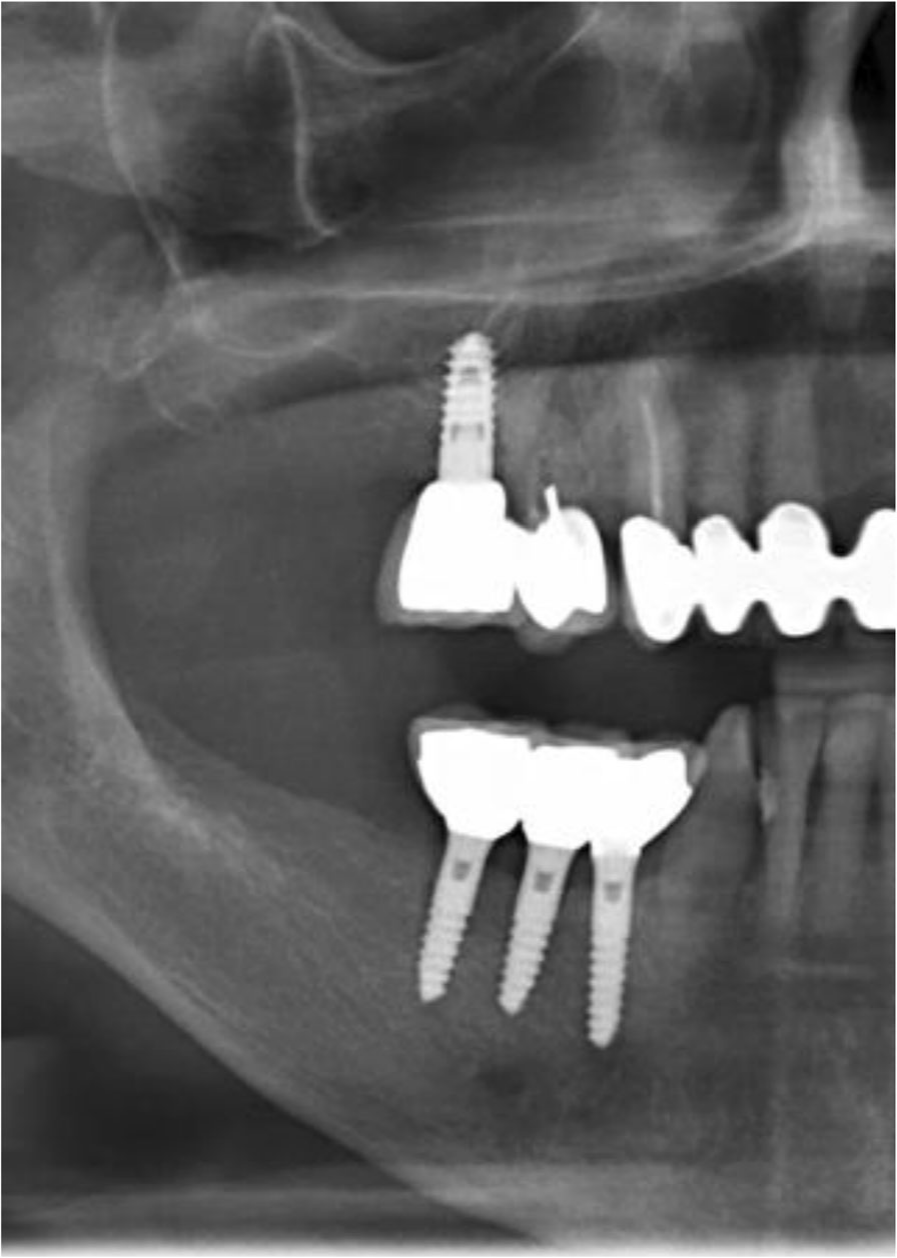
Radiological follow-up of the same patient 8 years post-OP

**Fig. 12:**
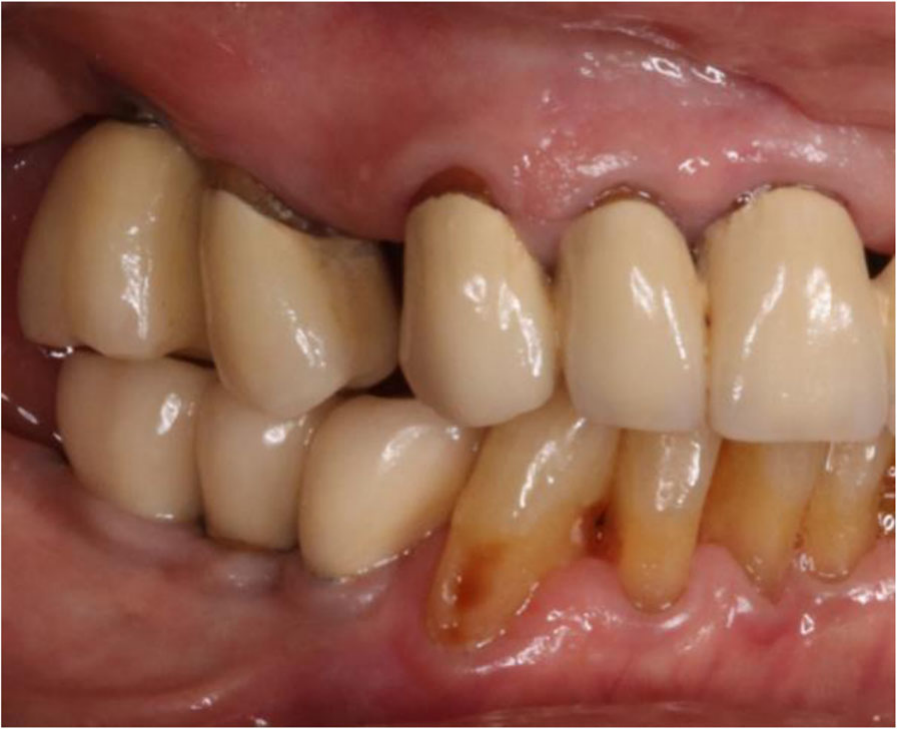
Clinical follow-up of the same patient 8 years post-OP. Please note the irritation-free keratinized peri-implant mucosa

Thirteen implants were provided with single crowns (25.0%), 24 implants with bridges, splinted crowns, respectively (46.2%), while 15 implants (28.8%) were used for the fixation of overdentures. All prosthetic reconstructions were screw retained. None of the patients reported prosthetic complications. Two clinical examples of MDI restorations in the anterior and posterior regions of the jaw are shown in the Figs. 5, 6, 7, 8, 9, 10, 11 and 12.

### Mean probing depth (MPD) and bleeding on probing (BOP)

Periodontal probing records of 29 implants (*n* = 12 patients) were available for analysis. Peri-implant probing depths were obtained by a six-point-measurement at each implant. Mean PD was 2.4 mm at the end of the mean clinical follow-up period, with a lower threshold value of 1.0 mm and an upper maximum value of 4.2 mm. No signs of suppuration were observed during probing the peri-implant sulcus in all implants. Bleeding on probing (BOP) occurred in 12 implants (41.4%), while 17 implants (58.6%) did not present any signs of bleeding. At implant sites with a positive BOP-Index, MPD was 2.7 mm, while MPD was 2.2 mm at the sites without any signs of bleeding. No significant difference in MPD could be observed between sites with positive or negative BOP (*p* = 0.059). No significant differences were observed between MPD in implants with/without augmentation measures (*p* = 0.145), augmentation material (*p* = 0.477), or prosthesis type (*p* = 0.208). Implants placed in the maxilla revealed significant higher MPD (3.1 mm) in relation to implants placed in the mandible (2.2 mm), whereas implant location within the respective jaw (anterior vs. posterior implant sites) had no significant impact on MPD (both 2.4 mm; *p* = 0.926).

## Recession

Recession was observed in 19 of the 29 analyzed implants (65.5 %). Mean overall recession was 0.5 mm, displaying measurement readings with a minimum value of 0.0 mm and a maximum value of 4.0 mm. Neither augmentation measures (*p* = 0.140), nor augmentation material (*p* = 0.409) had a significant impact on mean recession values. Type of prosthetic superstructure (*p* = 0.196) or healing modality (submerged/non-submerged) (*p* = 0.601) had no significant influence on mean recession as well. A significant negative correlation was observed between mean midfacial recession and mean crestal bone volume at reference point 1 (equicrestally) (*r* = − 0.376; *p* = 0.022) and reference point 2, 5.0 mm below level 1 (*r* = − 0.373; *p* = 0.023).

### Mean bone loss (MBL)

Peri-implant bone levels were measured in 49 implant sites. Overall peri-Implant MBL was 1.6 mm, and it was 1.6 mm as well, when radiological measurement values at the mesial and distal sites of the implant were analyzed separately. A statistically significant mean bone loss occurred at mesial and distal peri-implant sides, as measured at time of implant placement and at radiological follow-up (*p* < 0.001). Augmentation measures (*p* = 0.354), augmentation material (*p* = 0.094), and type of prosthetic superstructure (*p* = 0.052) had no significant influence on the amount of peri-implant bone loss. Implant location (maxilla/mandible) (*p* = 0.194) or location of implants within the respective jaw (anterior/posterior) (*p* = 0.972) had no significant impact on bone loss as well. In contrast, a clinical visible recession at least at one site of the implant had a significant impact on crestal bone loss (*p* < 0.001) (Table 1). Deeper recession defects and higher probing depths were significantly correlated to higher MBL (*p* < 0.001/*p* = 0.012), while there was no statistically significant difference in MBL between implants with a positive or a negative BOP-record (*p* = 0.059).

**Table 1:** MBL and prevalence of recession defects

Prevalence of recession	N	MBL	CI	STD	Significance ***p***
No recession	19	1.1	± 0.3	0.6	< 0.001
Recession	10	2.2	± 0.4	0.6	

A significant negative correlation was observed between the mean width of the alveolar crest, as calculated on the basis of available values, obtained by measurement on the three different crestal levels by CBCT, and the amount of overall peri-implant bone loss (*p* = 0.011) (Table 2). When considered separately, crest width on equicrestal level (level 1) had no significant impact on MBL, while lower crest widths on level 2 and 3 (5.0 mm/10.0 mm subcrestally) displayed a significant correlation to mean peri-implant bone loss. No significant correlation could be observed between age and MBL (*p* = 0.496).

**Table 2:** Correlation between MBL and alveolar crest width

Correlation between crest width and bone loss	Correlation coefficient r	Significance ***p***
Equicrestally (level 1)	− 0.099	0.255
5.0 mm subcrestally (level 2)	− 0.341	0.010
10.0 mm subcrestally (level 3)	− 0.322	0.014
Mean width	− 0.334	0.011

### Patient satisfaction

**Fig. 13:**
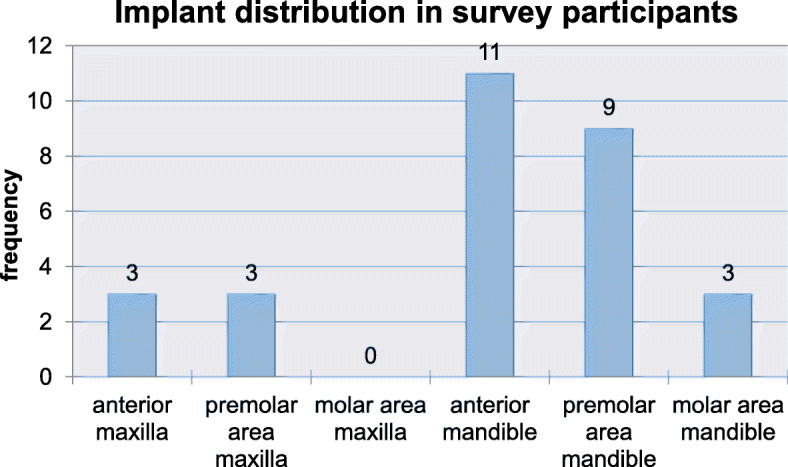
Implant distribution in the participants of the survey at the end of the follow-up period

Twelve of the 23 patients with in total 29 implants took active part in the survey. In these patients, the majority of implants (*n* = 20; 69.1%) was located in the anterior (*n* = 11; 38.0%) and premolar area (*n* = 9; 31.1%) of the mandible. The other nine implants were located in the anterior (*n* = 3) and the premolar area (*n* = 3) of the maxilla, and in the molar area of the mandible (*n* = 3) (Fig. 13). Most of the implants (*n* = 16) were used as bridge pillars, while eight implants were used for stabilization of full arch restorations. The remaining five implants were provided with single crowns in the anterior maxilla (*n* = 1), the anterior mandible (*n* = 3), and the premolar area of the mandible (*n* = 1). Ten different items had to be assessed based on a six-grade scale. Patients’ scoring displayed a high grade of satisfaction with treatment results and aesthetics.

Prevalence of recession (yes/no) had a significant impact on patients’ satisfaction with the clinical result after implant surgery and perception on implant aesthetics (*p* = 0.037). No significant correlation between the implant’s location and any parameter of PROMs could be found.

A significant positive correlation was observed between the extent of papilla height loss and midfacial recession, and the following items: surgical result, foreign body sensation, implant aesthetics, and recommendation of implant treatment. The better the tissue parameters, the better was the rating in the questionnaire concerning these four patient-related variables. Further significant positive correlation was assessed between a low extent of mesial and distal papilla height-loss and high estimation of surgical success, actual absence of complaints, and aesthetics of prosthetic treatment (Table 3).

**Table 3:** Significant correlation between specific PROMs and midfacial recession/loss of papilla height

	Location of recession					
	Midfacial gingiva		Mesial papilla		Distal papilla	
Parameter	***r***	***p*** value	***r***	***p*** value	***r***	***p*** value
Surgical result	0.500	**0.049**	1.000	**< 0.001**	0.738	**0.003**
Surgical success	0.389	0.105	0.817	**0.005**	0.632	**0.014**
Post-operative pain	− 0.310	0.163	− 0.310	0.163	− 0.209	0.256
Actually free of complaints	0.388	0.106	0.853	**< 0.001**	0.630	**0.014**
Foreign body sensation	0.500	**0.049**	0.500	**0.049**	0.738	**0.003**
Sensory disturbances	0.392	0.103	0.392	0.103	0.604	**0.019**
Implant aesthetics	0.500	**0.049**	1.000	**< 0.001**	0.738	**0.003**
Aesthetics of prosthesis	0.389	0.105	0.817	**< 0.001**	0.632	**0.014**
Would decide for implant treatment again	0.301	0.170	− 0.168	0.268	− 0.134	0.338
Recommendation of implant treatment	0.738	**0.003**	0.738	**0.003**	1.000	**< 0.001**

Both satisfaction with the surgical result and implant’s aesthetics after implant-prosthetic treatment were significantly affected by soft tissue conditions. Midfacial recession and loss of papilla height were significantly correlated with a worse grading in our questionnaire. In two of the three patients who rated aesthetics on implant-/prosthetic-level worse than grade 1, midfacial recession, and/or loss of papilla height between 2.0 and 4.0 mm had occurred in implants located in the anterior area of the maxilla and the mandible, thus being aesthetically challenging. Likewise, both participants, who displayed midfacial recession/papilla height-loss, rated surgical result and surgical success worse than grade 1. The third patient, who rated implant aesthetics worse than grade 1, did not display any loss of soft tissue.

## Discussion

Present study investigated survival, success, probing depths, signs of recession, and mean bone loss as clinical and radiological parameters after treatment of patients with severe horizontal alveolar bone loss (Figs.5, 6 and 7) with mini-implants using two different diameters and a two-piece design. Additionally, patients were asked to answer a self-developed questionnaire at the respective recall visit in our surgical center.

### Implant dimensions

Three implants were lost during clinical and radiological follow-up in our investigation, leading to a high survival rate of 94.2%. This finding stands in contrast to the results of a meta-analysis published in 2014 [19]. The results of the analyzed 16 studies displayed significant lower survival rates up to 75.0% of implants with a reduced diameter < 3.3 mm after a minimum clinical observation period of 1 year. The authors concluded that variables as type of prosthesis, implant surface, and timing of prosthetic loading had a significant impact on implant survival. Another systematic review reported a higher survival rate of 98.6% [20], while cumulative survival rates of another review were 94.7% after an observation period of 1 year, thus being in line with our findings, but not really comparable due to the markedly shorter follow-up period [21]. Results of another recently published systematic review yielded comparable results concerning implant survival rates/crestal bone loss between implants with standard or reduced diameter [6]. Mean bone loss around MDI ranged between 0.32 and 0.95 mm after a mean follow-up period of 3 and 5 years, thus being considerably lower than the mean bone loss of 1.6 mm in our study. MDI were defined as “narrow diameter implants” in present systematic review, and they were classified as implants with a diameter ≤ 3.3 mm. Missing classification and different designations are significant constraints in scientific literature on implant diameter and length, as displayed by the results of another systematic review [22]. A self-conducted literature search revealed a confusing variety of terms and dimensions for implants with reduced diameter. Implants with diameters between 1.8 and 2.4 mm [23, 24], < 3.0 mm [5], or 3.0 mm [25] were designated by different authors as “mini-implants.” Several other authors labeled implants with diameters of 3.0 mm [26], 3.0 to 3.5 mm [5, 27], < 3.3 mm [19, 28], 3.3 mm [29–33], ≤ 3.5 mm [34], or < 5.0 mm [35] as “narrow implants.” Other common designations for MDI were “small implants” (3.0 to 3.5 mm) or “diameter reduced implants” (3.3 mm). Hence, a consensus for a clear cut-off point between implants with a standard diameter and implants with a reduced, non-standard diameter is still missing.

### Clinical and radiological parameters

Besides missing standards in classification and designation of MDI, differences in outcome parameters due to the different characteristics of the implant’s neck design in one- and two-piece MDI had to be taken into account as well. The majority of the studies on MDI were performed with one-piece implants, while studies on implants with a two-piece design and a diameter ranging between 2.9 and 3.1 mm are still scarce. Hence, settlement of our experience with a two-piece MDI-system with similar investigations was not easily to perform.

Due to the scientific experience of the last 30 years, reasons for peri-implant bone loss around implants with standard diameters are not yet fully understood. Bone loss around implants seems to be dependent on factors like implant hardware, clinical handling, or patient characteristics [36]. With respect to the implant hardware, the position of the implant-abutment connection at the outer edge implant platform seems to play an essential role for the peri-implant hard- and soft-tissue health around the implant’s neck [37]. Reasons for crestal bone loss were traced back to biomechanical reasons like the stress concentration through movements in the microgap of the implant-abutment interface [38]. Another reason was supposed to be recumbent with a microbial leakage due to the large number of inflammatory cell infiltrates in the implant-abutment junction, probably promoting peri-implant bone loss through bacterial inflammation [39, 40]. Shifting the gap away from the peri-implant hard- and soft-tissues seemed to be a successful measure for better crestal bone preservation, as reported in many observational studies [41–49]. This concept was designated as “platform-switching” or “platform shifting.” As MDI in our investigation were not designed according to the platform-shift concept, the relatively high grade of crestal bone loss may be seen as a result of biomechanical and/or inflammatory processes at the implant-abutment interface, despite the reduced implant diameter. The low prevalence of clinically detectable signs of inflammation stands in contradiction to the assumption of a peri-implant inflammatory process as a cause for a higher crestal bone loss in our investigation.

According to the results of a Finite Element Analysis (FEA), no significant difference could be observed in average stress and strain at the implant’s neck in one-piece implants, two-piece implants with an external or internal connection design, respectively [15]. Results of a systematic review yielded less peri-implant bone resorption in two-piece implants with a wider diameter related to one-piece MDI after observation periods of 12 to 24 months [5]. Hence, presence of an implant-abutment interface, its position, the impact of the type of connection, and the collar design on hard- and soft-tissue parameters, as well as for the observed increased mean bone loss around implants, remain inconclusive for our investigation.

The observed significant correlations between the alveolar crest width at lower reference levels (levels 2 and 3), and the extent of vertical peri-implant bone loss, as well as crestal width at higher reference levels (levels 1 and 2), and the extent of mean midfacial recession may have served as indicators for a potentially high impact of alveolar crest width and the predictability of hard- and soft-tissue reaction after implant treatment. Thus, signs of hard- and soft-tissue resorption might be seen as a physiological effect, due to anatomical aspects concerning the alveolar crest width in some patient cases. Based on our results concerning the impact of alveolar crest width, a reduced bone volume at the upper two thirds of the alveolar crest might have had a significant impact on soft tissue recession, while a reduced crest width at the lower two thirds of the alveolar ridge might have had a significant influence on crestal bone loss.

A potential cause for the correlation between a reduced width in the lower two-thirds of the alveolar crest, might be potentially due to a reduced blood supply in bone, when implants with no appropriate width were placed, as stated in a narrative review [3]. While this may be used as explanation even when MDI were used in highly resorbed alveolar crests, a specific cause for this observation remains unexplained for the moment. For this reason, despite the fair clinical and radiological results, and the expanded opportunities of treatment with MDI, treatment planning should be performed with caution [3].

Clinical studies concerning the impact of alveolar ridge width on hard- and soft-tissue behavior are scarce. Two animal studies reported on the influence of the buccal bony crest width, reduced alveolar bony ridges, respectively, on hard- and soft-tissue changes [50, 51]. A significant higher mean vertical mesial/distal crest resorption of 1.5 mm/1.0 mm was observed in implants inserted in ridges with reduced alveolar crest width [50]. Our observations related to the significant negative impact of the alveolar crest width on the post-surgical behavior of peri-implant hard- and soft-tissues may be interpreted as a plea for a strict indication concerning the clinical decision for a minimally invasive treatment with MDI without extensive augmentation procedures.

### Patient-related parameters

We decided for a customized questionnaire, in order to aggregate information on self-perceived implant success with regard on the respective criteria of Buser et al. as well as on patient satisfaction in a single and short survey form. These criteria (except the fifth criterion “possibility of restoration”) were described as the most frequently reported variables on implant level in a systematic review by Papaspyridakos et al. [52], thus permitting a comprehensive overview in terms of implant-related success. The six-grade scoring was chosen in our questionnaire according to the German system of notation in schools, in order to facilitate estimation of the grade of satisfaction by the participants.

Missing validation, e. g., with pre-tests, may have acted as an additional potential source of bias in answering the questions of the different items of our questionnaire. For this reason, patients were supported by the same dental surgeon (LW) throughout the answering process, utilizing a standardized protocol, in order to minimize uncertainties or missing items, thus reducing risk for potential bias. As potential drawbacks of the dental surgeon´s presence during the answering process, interference on rating (with a tendency to better results) and a so called “interrogative suggestibility” could not be excluded as potential sources of bias [53].

Another major concern might have been the missing comparability of our results with the results of other investigations in this field of science, due to missing standardization in wording and/or scoring [54]. As presented by the results in our investigation, patients’ scoring demonstrated a generally high grade of satisfaction with clinical results and aesthetics after treatment with MDI, thus being in line with other investigations, comprising surveys on patient satisfaction after treatment with standard implants [55, 56] or with mini-implants [7]. The tendency for a poor rating of the aesthetical result due to soft-tissue loss was confirmed by a systematic review, showing a high correlation between the appearance of the peri-implant mucosa in the aesthetic zone and patient satisfaction [56].

## Conclusions

Treatment with MDI is a promising option for patients with a highly reduced alveolar ridge width. Due to the minimally invasive treatment modality, MDI seem to be very suitable especially for elderly patients, as demonstrated in this study. High implant survival and success rates as well as high patient satisfaction and suitability of this treatment option particularly in patients ≥ 60.0 years with reduced alveolar ridge width may serve as a reference for the predictability of treatment with MDI.

## Data Availability

The datasets used and/or analyzed during the current study are available from the corresponding author on reasonable request.

## Abbreviations

3D: Three-dimensional
BOP: Bleeding on probing
CBCT: Cone beam computer tomography
FDA: Federal Drug Administration
MBL: Mean bone loss
MDI: Mini-dental implants
MDP: Mean probing depth
PROMs: Patient-related outcome parameters
